# Addressing the Effects of War on Gaza’s Healthcare System

**DOI:** 10.7759/cureus.50036

**Published:** 2023-12-06

**Authors:** Sirwan K Ahmed

**Affiliations:** 1 Department of Adult Nursing, College of Nursing, University of Raparin, Rania, IRQ

**Keywords:** mental health, israel-palestine conflict, health consequences, gaza health care, gaza healthcare crisis

## Abstract

The protracted conflict in Gaza, marked by extensive Israeli attacks, has devastatingly crippled the healthcare system. Hospitals, clinics, and essential facilities have been directly targeted or damaged, leading to critical shortages in beds, equipment, and medications. The strain on healthcare workers has resulted in burnout, trauma, and mental health disorders. Moreover, repeated conflicts have triggered widespread psychological trauma among residents, exacerbated by a lack of mental health professionals and the stigma surrounding seeking help. International cooperation and humanitarian aid are essential to rebuild healthcare infrastructure, ensure the availability of medical supplies, and support mental health services. Additionally, diplomatic efforts for lasting peace in the region are crucial to mitigate the frequency and severity of conflicts, ultimately contributing to the stability and development of Gaza's healthcare system.

## Editorial

The prolonged conflict in Gaza has had severe and lasting impacts on healthcare [1]. Since October 7th, over 12,000 tons of explosives have been dropped, causing damage equivalent to the Hiroshima nuclear bomb. Israeli airstrikes destroyed 18 of the 35 hospitals, 32 primary care centers, and 11 water sanitation facilities. Attacks on healthcare facilities lead to 166 unsafe childbirths daily and result in the admission of 50 Palestinians, including children, per hour. Tragically, one child is lost every 15 minutes. Israeli airstrikes damaged 205 educational facilities, 69 healthcare centers, and 24 ambulances. Regrettably, 46 medical personnel lost their lives. There were over 1.4 million displaced people, 21 healthcare facilities experienced disruptions due to fuel shortages, and 1,400 people, including 720 children, are still unaccounted for under the rubble. About 70% of victims are children, women, and the elderly, and 50% of Gaza's families suffer from food shortages. The total death toll exceeds 7,700 people, with over 20,000 injuries, including 4,000 children and 1,750 women [2,3].

The wars in Gaza have led to the immediate and evident destruction of healthcare facilities [4], including hospitals, clinics, and medical supply centers, often targeted directly or damaged as collateral. This has left the fragile healthcare system in disarray, reducing its ability to provide care. Consequently, there's a shortage of beds, equipment, and medications, making it challenging for medical professionals to deliver essential services, leaving the wounded and sick without proper care and reducing their chances of survival.

Ongoing wars strain Gaza's healthcare system as health workers labor tirelessly amid hazardous conditions, treating casualties with limited resources. This leads to physical and emotional exhaustion, causing long-term issues like burnout, trauma, and mental health disorders. This adversely affects healthcare professionals' well-being and the quality of care. The Gaza blockade and damaged transport limit medical supplies, compounding the challenge of reaching those in need due to movement restrictions and limited border access. This worsens civilian suffering and strains healthcare providers, creating a declining public health situation with unmet medical needs.

War and conflict significantly affect the mental health of the population, as seen in Gaza [5]. Repeated wars cause widespread psychological trauma, anxiety, and depression among residents, with long-lasting effects due to ongoing violence and the loss of loved ones. Gaza's healthcare system is ill-prepared to handle this mental health crisis, due to a lack of professionals and the stigma around seeking help. International support and investment in mental health services are essential.

Addressing the challenges facing Gaza's healthcare system requires international cooperation and humanitarian aid [5]. The international community must play an active role in rebuilding healthcare infrastructure, ensuring the availability of medical supplies, and supporting mental health services. Additionally, diplomatic efforts to bring about lasting peace in the region are crucial to reducing the frequency and severity of conflicts, which will ultimately contribute to the stability and development of the healthcare system.
